# Challenges for Data Quality in the Clinical Data Life Cycle: Systematic Review

**DOI:** 10.2196/60709

**Published:** 2025-04-23

**Authors:** Doyeon An, Minsik Lim, Suehyun Lee

**Affiliations:** 1 Department of IT Convergence Graduate School Gachon University Seongnam Republic of Korea; 2 Department of Computer Engineering College of IT Convergence Gachon University Seongnam Republic of Korea

**Keywords:** clinical research informatics, data quality, data accuracy, electronic health records, frameworks, quality of health care

## Abstract

**Background:**

Electronic health record (EHR) data are anticipated to inform the development of health policy systems across countries and furnish valuable insights for the advancement of health and medical technology. As the current paradigm of clinical research is shifting toward data centricity, the utilization of health care data is increasingly emphasized.

**Objective:**

We aimed to review the literature on clinical data quality management and define a process for ensuring the quality management of clinical data, especially in the secondary utilization of data.

**Methods:**

A systematic review of PubMed articles from 2010 to October 2023 was conducted. A total of 82,346 articles were retrieved and screened based on the inclusion and exclusion criteria, narrowing the number of articles to 851 after title and abstract review. Articles focusing on clinical data quality management life cycles, assessment methods, and tools were selected.

**Results:**

We reviewed 105 papers describing the clinical data quality management process. This process is based on a 4-stage life cycle: planning, construction, operation, and utilization. The most frequently used dimensions were completeness, plausibility, concordance, security, currency, and interoperability.

**Conclusions:**

Given the importance of the secondary use of EHR data, standardized quality control methods and automation are necessary. This study proposes a process to standardize data quality management and develop a data quality assessment system.

## Introduction

As data continue to accumulate, the question of how to use neglected data has received increasing attention. In particular, the need for quality control in the use of electronic health record (EHR) data has been emphasized. EHR data are expected to facilitate the development of national health policy systems and provide useful information for improving public health and medical technology [1]. As the current clinical research paradigm shifts to one of data centricity, the use of EHR data has increasingly been emphasized [2].

The quality of EHR data research depends on the quality of the generated data, which is a major research limitation. EHR data are essential in preclinical research, which is conducted to study the future of diseases and draft policies. Therefore, integrated data must be used seamlessly and incorporate different types of data. Currently, various methods for integrated data management are being developed [3-9], but quality control standards are set differently for each data type, and discussions in this regard are challenging because of the nature of EHR data [10-13].

Although research into EHR data quality management is actively underway, a gold standard for assessing data quality remains absent. Inconsistencies in data formats and terminology, a lack of standardization, security issues, and challenges in processing large-scale data persist as major obstacles to establishing standardized EHR data management practices [14,15]. Another critical challenge in EHR data management is achieving consistency across data sets from different hospitals and health care systems [16]. The variability in data collection methods and formats among institutions complicates the integration of data sets, undermining the reproducibility and reliability of research [17].

The consistent quality of EHR data is a critical factor in the performance of data analytics. Meeting data quality standards requires a management system that is appropriate for each stage of the data life cycle [18,19]. However, no standardized approach is available to assess the quality of EHR data [14]. For accurate and consistent research on EHR data, common data models (CDMs) such as the Observational Medical Outcomes Partnership CDM and Sentinel CDM are being built [20,21]. However, CDMs are evaluated individually depending on their type [22-24].

The quality of clinical data depends on the quality of the data on which they are built, and such dependence is another major research limitation. A data quality management process defines the basic principles of data management and enables accurate, consistent control of data quality [25]. High-quality data can be defined as such when they are not built piecemeal but are managed throughout the entire process of operation and use.

This study aimed to understand the importance of clinical data quality management and the life cycle–based clinical data quality management process. Accordingly, the existing literature on EHRs and clinical data quality was reviewed, and the guidelines for the predefined clinical data quality management processes of planning, implementation, operation, and utilization [26] were subsequently considered.

## Methods

### Definition of the Clinical Data Life Cycle

In the context of systematic data quality management, we defined the life cycle of clinical data quality management [26] as the quality management activities for health care data that include a series of steps from data construction to operation and use [26].

### Literature Review on Data Quality

We aimed to identify articles that extensively discussed the generation and quality of EHR data. In this study, an EHR refers to all electronically stored records of patient health information, encompassing both electronic medical records and personal health records. To conduct the literature review, we followed the methods of previous studies that closely reviewed previous EHR data [14,27-29]. A PubMed literature search was conducted by the first author in October 2023. The keywords for the search were text words and Medical Subject Headings such as “data quality,” “data accuracy,” “quality indicators,” “quality of health care,” “quality control,” and combinations of these terms (Textbox 1). The literature search was limited to articles published in English.

**Search terms.**

'quality[ti]' AND (‘data quality’ OR ‘data accuracy’ OR ‘Quality of Health Care’ OR ‘Quality Indicators’ OR ‘quality control’) AND (EHR OR electronic medical record OR computerized medical record OR medical records systems, computerized [mh]) AND English[lang] NOT (review OR Clinical Trial OR Documents OR Books)

A total of 82,346 articles were retrieved from PubMed. To select articles suitable for our research purpose, we referred to previous studies and applied the inclusion and exclusion criteria listed in Textbox 2 [14,27-29]. The studies were evaluated based on their relevance to the assessment and management of data quality of EHR data. This was done by applying inclusion and exclusion criteria to the titles and abstracts of the studies. This process was conducted by an author with a degree in public health (DA) and cross-checked by another author specializing in health informatics (MS) to minimize bias. In cases of disagreement in study selection, final decisions were made through thorough discussion. A total of 851 articles were selected after the first review. In the second review, all articles were manually reviewed by the first author to ensure they met the criteria. Subsequently, all papers related to data quality were selected and classified based on the following 4 keywords: “data quality,” “EHR assessment,” “treatment quality,” and “hospital quality.”

**Inclusion and exclusion criteria.**

Inclusion criteria

- Original research using data quality assessment methods
- Focus on data derived from electronic health records or related systems

Exclusion criteria

- Guidelines limited to one medical area (eg, cardiology) without generalization to other areas
- Review papers
  Guidance aimed at governing bodies
  Published before 2010
  Papers not in the English language
  No full text available
- Not a paper on data quality issues

To focus on data quality management for clinical data analysis, we reviewed the full text of each article containing 2 of the 4 keywords, that is, “data quality” and “EHR assessment.” In this process, we reviewed medical data quality and 13 relevant guidelines. Ultimately, 105 studies were included.

For each article, we described the category, definition of data quality, data quality management methods, and quality control procedures. The literature categories included the main perspectives, research methods, and research findings. For efficiency, we reviewed the articles by classifying them into the following 4 topics: “framework,” “quality measures,” “quality tool,” and “interview.” Framework papers included articles addressing general procedures for data quality, while papers on quality measures included those involving data evaluation. Articles on quality tools included those that developed data evaluation tools, while interview articles included those that evaluated data based on the opinions of experts in actual hospital settings.

We abstracted the general methods and procedures for data quality management based on data life cycle and evaluation methods in each paper. To establish standards for the data life cycle, we analyzed the literature related to data frameworks and identified ways to construct data quality management procedures. The data quality evaluation criteria, quality evaluation methods, data types, and vocabulary used in each article were also collected. The content of the articles was then repeatedly reviewed to define their quality control dimensions.

To organize the overall data quality assessment methodology, we reviewed the literature that mentioned the data life cycle; however, finding articles offering a clear definition was difficult. Data quality must be consistently defined [30]. The literature shows how clinical data are constructed and evaluated according to different processes. Studies have been conducted to define methods for evaluating data; however, the series of processes through which data are generated and used has not been considered. We realized that consistent data quality management could be implemented by identifying and defining the data characteristics highlighted in the literature. Our study attempted to define a set of processes through which data are constructed, operated, and used through a literature review and to include all commonly occurring concepts. We then reviewed all articles to collect data on the use of the newly defined processes and dimensions.

## Results

### Data Quality Assessment Framework Based on the Clinical Data Life Cycle

Data quality can be defined as “the level that can continuously meet the various activity purposes or satisfaction of users using data” [31]. Data quality management refers to a set of activities that ensure data quality. With the goal of developing and implementing high-quality data, data quality management encompasses all data-related management activities, from data creation to use [26].

**Figure 1:**
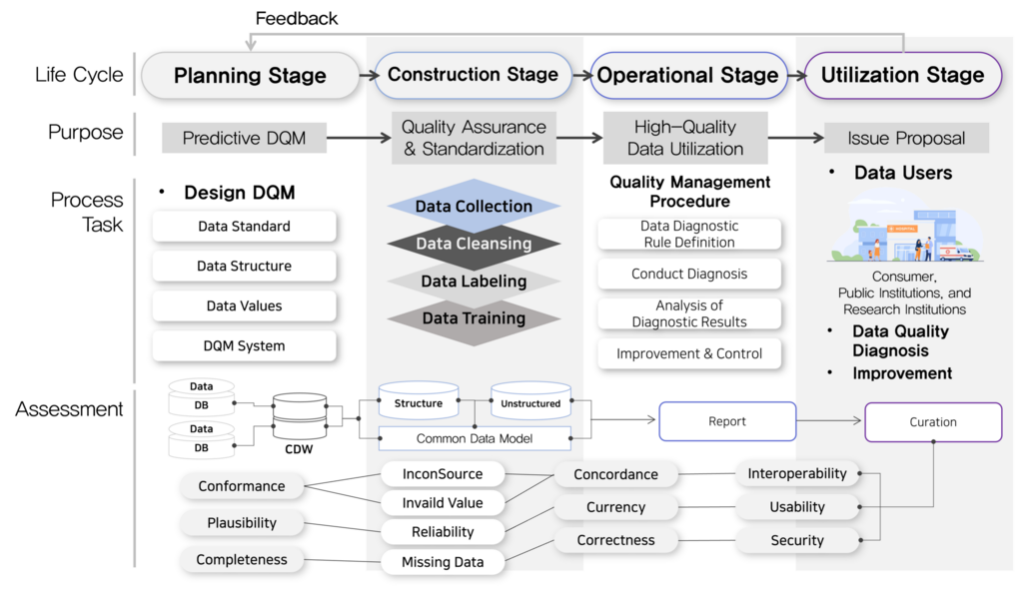
Life cycle of clinical data quality management (DQM). CDW: common data warehouse; DB: database.

Figure 1 illustrates the life cycle of clinical data and defines the data quality management methods according to the life cycle stage. We used the clinical data life cycle, which consists of the planning, construction, operation, and utilization stages [26]. In producing high-quality data, data must be managed according to the data life cycle and governance principles [26].

We established the definitions for each clinical data life cycle stage by reviewing the literature (Table 1). The literature included in the review often described the data life cycle for improving hospital EHR quality, quality measurement, and clinical decision support [32-36].

**Table 1:** Defining the life cycle of clinical data quality management.

Life cycle stage	Definition	References
Planning stage	Defining data standards based on the direction of data and creating a clear strategy for establishing quality management activities	[6,32,33,37]
Construction stage	Considering the characteristics among data sets, collecting data, and proceeding with overall data construction and management that reflect clinical attributes	[32,37-43]
Operation stage	Conducting data quality assessments on the constructed data and reviewing them from various angles and perspectives	[32,33,37,44,45]
Utilization stage	Sharing the outcomes of data quality validation, implementing data quality enhancement activities, and recalibrating the overall data quality	[32,33,37,46]

### Planning Stage

In the planning stage of data quality management, key issues such as the data to be generated and their documentation and organization, storage and security, stewardship, and accessibility for reuse and sharing are considered [47]. Developing a data management plan should involve describing how data will be handled throughout the life of the project and after completion and establishing principles that are easy to implement [48].

### Construction Stage

The construction stage involves quality control. It is also called the big data life cycle stage [25] (Figure 1). This data life cycle stage consists of 4 stages: data collection, data cleaning, data labeling, and data learning. At each stage of the life cycle, the tasks to be performed vary. For example, data quality control standards must be established and reflected in the data collection stage.

### Operation Stage

Managing constructed data is the most active phase of data quality management. When building quality data, quality control must be implemented starting from the planning stage. However, not all data are built with quality control in mind from the planning stage. In data quality management, the operational stage involves activities to diagnose and improve the quality of the data loaded in data construction projects.

### Utilization Stage

The main users of public medical data are public institutions and research institutes. Data quality management organizations must continuously implement improvements to provide high-quality data by adhering to the requirements of both data providers and consumers. Moreover, data must be continuously and accurately managed to provide high-quality medical services [9]. Accordingly, a support system must be institutionalized to continuously communicate with researchers on the use of medical data, and a foundation such as medical data standards must be established to ensure the uninterrupted provision of high-quality data.

### Proposed Data Framework Based on the Clinical Data Life Cycle

In our literature review, we found one commonality: All stages are interrelated and emphasize the need to manage data from a holistic, life cycle perspective [26]. The plan-do-study-act (PDSA) cycle, which was frequently mentioned in most of the articles we reviewed, is primarily used for short-term processes, such as data construction or operation [33,38,46]. Therefore, the PDSA cycle, which is mainly used in the data construction stage, could not be applied in our study. The clinical data life cycle proposed in this study is designed to manage data comprehensively from a governance perspective. It is structured in a mutually organic manner, allowing for the reapplication of improvements after EHR data planning, construction, and secondary use. A set of procedures, such as the data framework, provides an environment for researchers to understand data, identify quality issues, and address them effectively [49]. As data significantly influence research outcomes, they must meaningfully be evaluated and managed throughout their life cycle [30]. Some studies did not consider data from a life cycle perspective [34,35,50-52]. Nevertheless, they considered the ecological use of data. They also considered the impact of data on hospital treatment processes [34,35]. Thus, data operations are organically linked, reflecting the interplay between different stages.

### Dimensions of the Data Life Cycle and Clinical Data Quality Management

**Figure 2:**
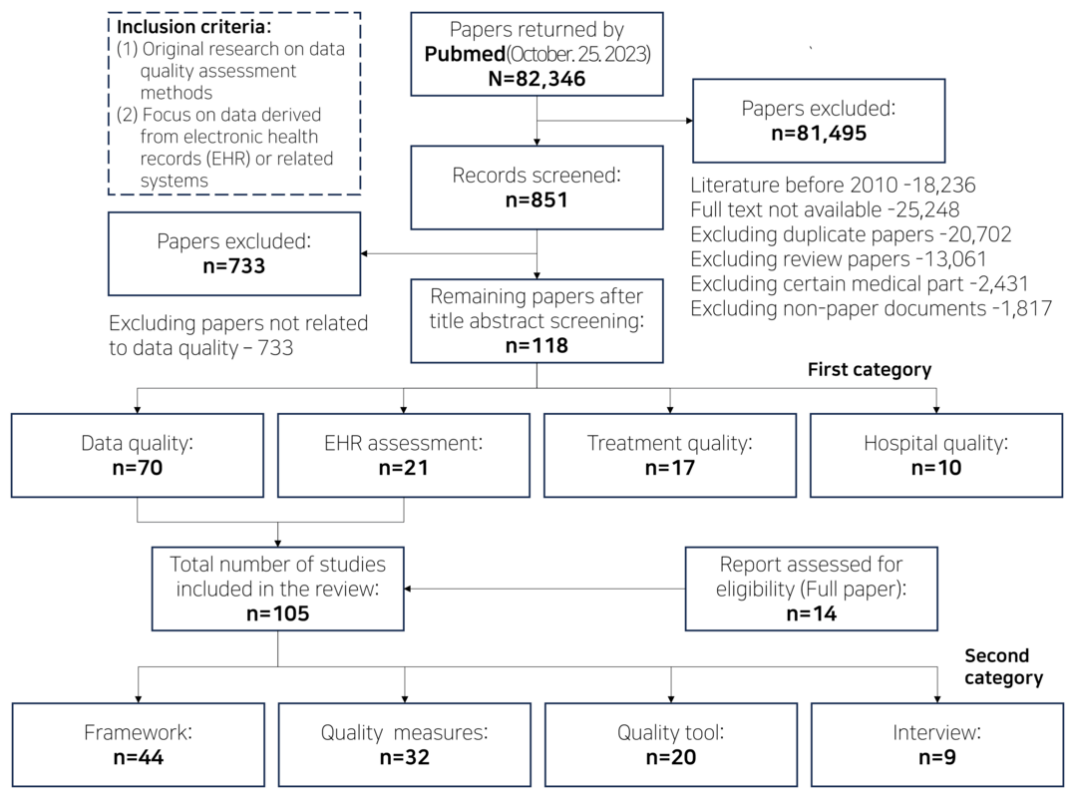
Diagram of the literature review process for clinical data quality management.

**Figure 3:**
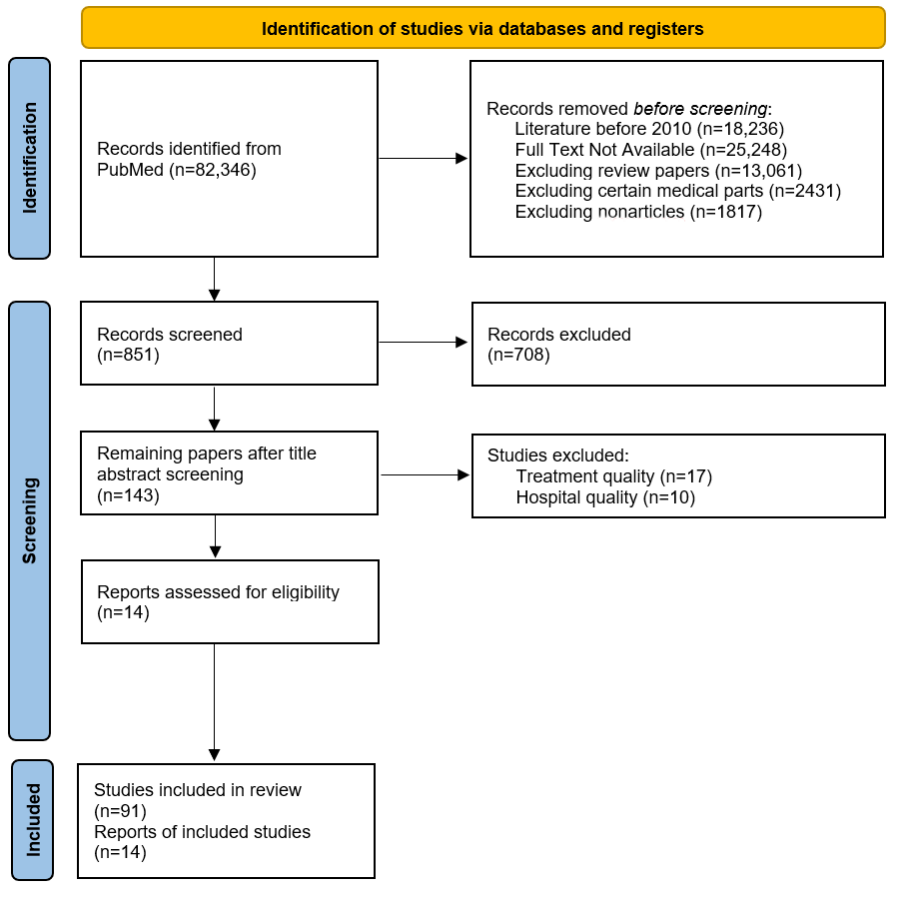
PRISMA (Preferred Reporting Items for Systematic Reviews and Meta-Analyses) 2020 flow diagram.

The set of reviewed papers comprised 44 papers on data framework, 32 papers on quality measures, 20 papers on quality tools, and 9 papers on interviews (Figures 2 and 3; Multimedia Appendix 1). Completeness was identified as the most commonly used indicator, particularly in 94 papers (Table 2 and Table 3). Research using data quality dimensions can be classified according to the stage of the clinical data life cycle, with the greatest amount of research occurring in the planning and implementation phase (Table 3).

**Table 2:** Definitions of the life cycle of clinical data quality management and dimensions of data quality.

Dimension	Definition	Synonyms
Completeness	Assessing the extent to which data have been fully constructed in accordance with their characteristics and intended design	Completeness, correctness, conformance, incompleteness, consistency
Plausibility	Degree of reliability in data values and the significance of the associated information	Accuracy, consistency, relevance
Concordance	The extent to which data can be stored in accordance with their characteristics based on standards	Structure, standardization
Security	The extent to which data are trustworthy and accessible only to authorized users	Security, availability, confidentiality, representation, confidentiality, trustworthiness
Currency	The extent to which data can be provided promptly when needed	Currency, timeliness, currentness
Interoperability	The degree to which data operation is flexible, providing a sufficient and useful level of information that satisfies users	Availability, manageability, variability

**Table 3:** Life cycle of clinical data quality management and dimensions of data quality.

Dimension	Planning stage (n=69)			Construction stage (n=99)			Operation stage (n=95)			Utilization stage (n=72)	
	Mentions, n (%)^a^	Articles	Mentions, n (%)^a^		Articles	Mentions, n (%)^a^		Articles	Mentions, n (%)^a^		Articles
Completeness (n=107)	22 (20.6)	[6,7,18,19, 25,32,33,43,45,53-65]	34 (31.8)		[7,9,18 ,19,25,32,39 ,40,43 ,45,53-77]	30 (28)		[7,9,15 ,19,25,32,34 ,43,49,50,55-57,59 ,63,65,70,72 ,75,76,78-85]	21 (19.6)		[7,16,18,19,22,25,32,43,49 ,50,56,63,65-67,75,78,84-87]
Plausibility (n=72)	19 (26.4)	[6,7,11,17-19 ,25,32,33,43 ,45,51,54,56,61,63-65,88]	25 (34.7)		[7,9,11,17,19,22,25,43,45 ,51,54-56,61,63-66,68-70 ,75,76,88,89]	26 (36.1)		[7,9,11 ,15,17,19,25,43,45 ,46,49 ,51,56,63,65,70,75 ,76 ,79-83,88,90,91]	19 (26.4)		[7,11,16-19,25,32,43 ,46,49,56,65,66,75,86,88,90]
Concordance (n=81)	18 (22.2)	[6,7,17-19,25,32,33,43,45 ,51,56,57,59,62,63,65]	22 (27.2)		[7,9,17 ,19,25,43-45 ,51,55-57,59 ,62,63,65,67 ,70,75,76]	23 (28.4)		[7,9,17,19,25 ,32,43,44,49,51,56,57,59 ,63,65,70 ,75,76,79 ,80,85,90]	18 (22.2)		[7,16-19 ,25,32,43,49,56,63 ,65,67,75,85,86,90]
Security (n=33)	8 (24.2)	[17,19,25 ,32,45,51,58 ,60,63]	9 (27.3)		[17,19,25,45,51 ,58,60,63,89,91]	7 (21.2)		[17,19,25,51,63,79,90,91]	9 (27.3)		[16,17,19,25,32,63,86,87,90]
Currency (n=42)	9 (21.4)	[33,43,45,52,54 ,57,62,63,92]	14 (33.3)		[11,15,17,43,52,55,57 ,62,63,67,71,72,92,93]	10 (23.8)		[11,15,17,43,55 ,57,63,72,79,85,93]	9 (21.4)		[11,16,17,32,43,57,63,67,85,86]
Interoperability (n=35)	7 (20)	[17,33,35,36,62,63,94]	8 (22.9)		[17,35,36,46,55 ,63,74,79,80,94]	10 (28.6)		[17,35,36 ,46,55 ,63,74 ,79 ,80,94]	10 (28.6)		[17,32,35,36,46,63,75,86,90,94]

^a^Distribution of each dimension across the stages of the clinical data life cycle (planning, construction, operation, and utilization), calculated as a proportion of each dimension’s total.

#### Completeness

Completeness was mainly used in the construction or operation stage and was used as an indicator for EHR evaluation [66,67], data quality system development [7,53,78], data recognition [17], and comparative evaluation [50]. The related terms used in the articles included correctness, conformance, incompleteness, and consistency.

#### Plausibility

Plausibility was the second most frequently used indicator, with 72 references mentioning it. It was often used in data evaluation during the operation phase of the data life cycle. It was mainly mentioned in the literature on data tool development [54,55], framework presentation [45,68], data measurement [69], and data quality assessment [7,66,89].

#### Concordance

Similar to completeness and plausibility, concordance was frequently mentioned in the construction and utilization stages. Concordance can be considered an indicator that determines whether the characteristics of different data are best expressed and stored based on standards. Concordance was mentioned in the studies that developed, experimented with, and evaluated quality management tools [9,51,54,56,57,70,90]. The related terms mentioned in the articles included structure and standardization.

#### Security

As EHR data are sensitive, great attention must be paid to ethical issues and data leakage. Therefore, the security of EHR data is crucial. In contrast to the aforementioned 3 indicators, which reflect the completeness of data, security was most frequently mentioned in the construction and utilization stages. The related terms mentioned in the articles included availability, confidentiality, representation, and trustworthiness.

#### Currency

Currency was mentioned most often during the data construction stage. In particular, the availability of data must be determined during data construction. Having readily available data is critical for the research process. The terms representing currency included timeliness.

#### Interoperability

The most cited limitation of EHR data is the difficulty with linking data between hospitals. By combining and sharing data already in use, more resources can be utilized. The indicator representing this relation is interoperability. The literature review in this study revealed a strong emphasis on interoperability, but it was not mentioned in articles defining other data quality indicators.

## Discussion

### Principal Findings

This study reviewed the existing literature, focusing on the importance of quality management from the EHR data life cycle perspective. Accordingly, an EHR data life cycle framework was defined, and 6 quality indicators were identified.

Data quality ensures the validity of research findings and provides information to demonstrate the appropriateness of EHR data use [49]. In this study, we identified the requirements for each stage of the data life cycle, including cycle-specific objectives, tasks, and evaluation metrics, to determine the validity of data. Data quality is a fundamental element for determining whether data have been constructed for their intended purpose [95]. Quality management must be applied at every stage of data processing to ensure that all data are reliable and appropriately handled [96].

The metrics identified in this study were frequently mentioned in the literature. We mapped the categories proposed in this study for currency and interoperability, which differ from the indicators proposed in previous studies. An accurate definition of these dimensions is essential for data quality. The definition of completeness alone can vary the completeness ratio of data depending on the type of data or the purpose for which quality is defined [28,86]. Dimensions have been developed to clearly define and automatically measure data [45]. Currency and interoperability metrics are not entirely new. They were mentioned repeatedly in various studies [33,43,45,52,54,57,62,63,92]. Currency refers to information about current data [63] and is primarily used for temporal information when representing the lifetime of data [16]. Temporal factors exert a significant effect on research results. In addition, currency should be considered when visualizing data quality results [42].

This study proposes a total of 6 data quality dimensions based on a comprehensive literature review. These indicators are not universally applicable across all data sets; additional dimensions may be warranted depending on specific conditions (Multimedia Appendix 2). For instance, bias can emerge based on data construction or the research environment. Addressing bias is crucial and has been emphasized in numerous studies on data quality [14,16,83]. In this regard, assessing task relevance is vital to verify that the constructed data meet their intended objectives and are effective for their purpose [45]. Furthermore, if data are integrated from multiple sources rather than generated from a single system, it is critical to evaluate consistency across data sets using the variability dimension [57]. In clinical settings, the validity and reliability of data are fundamental to the development of safe and accurate predictive models [57]. It is also necessary to assess usability to confirm that researchers in clinical environments can use data both effectively and efficiently [7,42] (Multimedia Appendix 3). Before using and measuring any data quality dimension, the purpose and research objectives of the data must be thoroughly understood, and the indicators must be selected accordingly. Systematic data quality assessments are essential at each phase of the data life cycle to ensure comprehensive data utilization. Each dimension can play a vital role in ensuring data accuracy, reliability, and efficiency, thereby enhancing the reproducibility and validity of the research. Developing a well-defined data quality plan minimizes unnecessary processes and costs and directly enhances data transparency and trustworthiness.

The majority of discussions on the quality of EHR data have centered on 3 key areas: conformance, plausibility, and completeness [6,42,49,70,86]. However, the actual quality of data can vary significantly depending on the measurement methods and management strategies used, due to factors such as the type and volume of data, data construction environment, characteristics of the disease, and type of system in which the data are generated [75,94]. A substantial body of research has proposed and developed a multitude of indicators. Through a comprehensive review of the literature, we identified that dimensions such as accuracy, consistency, completeness, and currency are closely interrelated according to data characteristics. Additionally, these indicators may vary in relevance depending on the data life cycle stage. Many studies, however, have overlooked these aspects. Recognizing the interdependence between dimensions while accounting for the unique characteristics of the data is crucial to establishing high-quality data.

When ensuring effective data quality management, simplified data guidelines that can be easily applied must be considered. Data quality management frameworks and guidelines are being developed in a data-specific manner [12,18,19,25,65]. From the data life cycle perspective, data quality management must be coordinated from a governance perspective throughout the entire life cycle. Several different types of data exist. To actively manage the quality of different data, more diverse data quality management methodologies must be developed [97]. Meanwhile, ensuring that data are usable and consistent requires clearly targeted and planned quality control procedures [48]. Regarding ensuring the scalability of data connections, quality control for integrated data using standardized procedures should be implemented from the planning stage [98].

In our study, we emphasized the importance of interoperability in the use of EHR data. The use of EHR helps researchers conduct their studies involving large amounts of data at a low cost [99] and facilitates the analysis of health information from thousands of individuals. Ideally, EHRs should be accurate and complete because they contain all health records [100]; however, EHR data face numerous quality issues [4,101]. In addition, challenges arise from the use of different EHR systems across hospitals and the heterogeneity of data, resulting in limited interoperability. Limited interoperability and inconsistent data exchange across settings are significant barriers to quality improvement [102]. The interoperability of EHRs with medical data is becoming increasingly valuable because of its potential to exponentially increase the availability of data or directly impact the activation of research. EHR systems can efficiently support data structuring and quality measurement results and have a great impact on patients and their time [102]. Interoperability among EHR systems refers to the linking of data, which improves data usability. Therefore, regulating the data structure or transfer standards between systems is essential to improve data quality and interoperability.

Considerable effort has been made to improve the quality of EHRs. These efforts include the development of automated data quality assessment systems [9,42,103], organization of quality indicator events, and development of metrics. Data must be sufficiently flexible to be used for multiple purposes. Moreover, data must be managed according to user needs, and diagnoses must be made based on the users’ purpose. When producing high-quality data, the data must be thoroughly examined from a data life cycle perspective, starting from data construction, to ensure that data standards are well established and applied, data are consistently secured, and errors are minimized [104].

Establishing criteria for data quality is critical because the data sources for research questions represent a major determinant of research outcomes. Several factors necessitate the establishment of data quality standards. First, the types of data required vary according to the research topic, and data types and structure are significantly diverse. In addition, medical practices and health care systems vary widely worldwide, and their differences can affect the relevance of data to research questions [12]. Data must be managed continuously and accurately to provide high-quality medical services [9]. Consequently, the perspectives for measuring the level of data quality must be defined, and the criteria for what should be measured must be established [25].

Investing in EHR data quality management improves clinical outcomes [34]. As hospital resources are limited, data preprocessing and quality assessment must be automated to avoid wasting resources. Many hospital researchers have focused on automating data quality assessment [3,6,8,9,59,77,105]. However, automation across all data sets lacks a unified standard, and different tools have been developed for different data types and languages. Given the diverse criteria and forms of EHR data, such approaches are not pragmatic [14]. Accurately defining the domains and task ontologies for measuring data quality in the automation process is critical [45,59]. Various methodologies and quality criteria have been identified [29]. Nevertheless, flexible tools that consider interoperability must be developed, and existing methodologies must be used to create a unified automation tool [14].

### Limitations

Our literature review has several limitations that need to be considered. First, the literature selection was conducted solely by the first author, which may introduce subjectivity to the process and result in classifications that other reviewers might not agree with. Although cross-review efforts were made, the lack of a multireviewer approach may limit the generalizability of the findings. Second, in this study, we conducted the literature search using only one database. Due to the use of a single source, there may be a risk of missing other relevant studies. However, prior to conducting our study, we performed the same search in other databases and observed similar results to those obtained from PubMed, the database ultimately used in this research. Third, the quality dimensions identified in this review, derived solely from existing literature, have not been validated by clinical experts. The absence of expert validation may limit the practical applicability of these dimensions in clinical settings, indicating a need for further expert review.

### Conclusion

As the value of EHR data increases, the demand for high-quality data also rises. Standardized quality management and automation of data quality assessment are necessary to produce high-quality data and improve their usability. This study focuses on the secondary use of EHR data, reviews the existing literature, and redefines quality management indicators from a data life cycle perspective. As data quality assessment methods based on the data life cycle perspective have not yet been developed, future work should focus on developing data quality assessment systems with an emphasis on standardized frameworks and tools that consider the specific characteristics of the data. 

## Appendix

Multimedia Appendix 1 Data Search List.

Multimedia Appendix 2 Additional Quality Dimension.

Multimedia Appendix 3 Term of Data Quality Management.

Multimedia Appendix 4 PRISMA (Preferred Reporting Items for Systematic Reviews and Meta-Analyses) 2020 checklist.

## Abbreviations

CDM: common data model
DQM: data quality management
EHR: electronic health record
PDSA: plan-do-study-act

## Data Availability

The data supporting this article are available upon request from the corresponding author.
