# Supplementary material for: Challenges for Data Quality in the Clinical Data Life Cycle: Systematic Review
Source: J Med Internet Res. 2025 Apr 23;27:e60709. doi: 10.2196/60709 (PMC12059509; doi:10.2196/60709)
Supplement: Multimedia Appendix 2 [file jmir_v27i1e60709_app2.docx]

Multimedia Appendix. Additional Quality Dimension

| Dimension | Definition | |
| --- | --- | --- |
| Bias | This data quality issue arises when specific data points within electronic health record (EHR) data are absent or incomplete, due to systematic rather than random omissions. Such instances may be attributed to information bias or measurement bias. | [1-3] |
| Domain Consistency | An indicator of how consistently EHR data is managed using standardized data definitions and formats within a specific domain (e.g., cancer research, emergency medicine). | [4] |
| Duplication | The occurrence of events that are, in fact, physically possible to occur only once. | [5] |
| Feasibility | An assessment of whether a specific data quality metric or improvement system is practical and achievable within a real clinical setting to meet its intended objectives. | [6] |
| Maintainability | The extent to which information can be maintained to achieve the intended improvements (e.g., through modification, revision, updating, adjustment, and upgrading). | [7] |
| Task Relevance | This is a metric for assessing the effectiveness of data design in achieving the objectives of a specific research task or analytical purpose. | [4] |
| Usability | A concept for evaluating whether end-users can utilize data effectively and efficiently. | [8, 9] |
| Variability | An indicator that evaluates the consistency in recording and managing data collected from two or more EHR systems. | [10] |

1. Lewis AE, Weiskopf N, Abrams ZB, Foraker R, Lai AM, Payne PRO, et al. Electronic health record data quality assessment and tools: a systematic review. Journal of the American Medical Informatics Association. 2023;30(10):1730-40. doi: 10.1093/jamia/ocad120.

2. Wang H, Belitskaya-Levy I, Wu F, Lee JS, Shih M-C, Tsao PS, et al. A statistical quality assessment method for longitudinal observations in electronic health record data with an application to the VA million veteran program. BMC Medical Informatics and Decision Making. 2021 2021/10/20;21(1):289. doi: 10.1186/s12911-021-01643-2.

3. Kahn MG, Raebel MA, Glanz JM, Riedlinger K, Steiner JF. A pragmatic framework for single-site and multisite data quality assessment in electronic health record-based clinical research. Med Care. 2012 Jul;50 Suppl(0):S21-9. PMID: 22692254. doi: 10.1097/MLR.0b013e318257dd67.

4. Johnson SG, Speedie S, Simon G, Kumar V, Westra BL. Application of An Ontology for Characterizing Data Quality For a Secondary Use of EHR Data. Appl Clin Inform. 2016;7(1):69-88. PMID: 27081408. doi: 10.4338/aci-2015-08-ra-0107.

5. Wang Z, Talburt JR, Wu N, Dagtas S, Zozus MN. A Rule-Based Data Quality Assessment System for Electronic Health Record Data. Appl Clin Inform. 2020 2020/09/23;11(04):622-34. doi: 10.1055/s-0040-1715567.

6. Dy SM, Lorenz KA, O'Neill SM, Asch SM, Walling AM, Tisnado D, et al. Cancer Quality-ASSIST supportive oncology quality indicator set. Cancer. 2010;116(13):3267-75. doi: <https://doi.org/10.1002/cncr.25109>.

7. Fadahunsi KP, Wark PA, Mastellos N, Neves AL, Gallagher J, Majeed A, et al. Assessment of Clinical Information Quality in Digital Health Technologies: International eDelphi Study. J Med Internet Res. 2022;24(12):e41889. PMID: 36472901. doi: 10.2196/41889.

8. Engel N, Wang H, Jiang X, Lau CY, Patterson J, Acharya N, et al. EHR Data Quality Assessment Tools and Issue Reporting Workflows for the 'All of Us' Research Program Clinical Data Research Network. AMIA Jt Summits Transl Sci Proc. 2022;2022:186-95. PMID: 35854725.

9. Kapsner LA, Mang JM, Mate S, Seuchter SA, Vengadeswaran A, Bathelt F, et al. Linking a Consortium-Wide Data Quality Assessment Tool with the MIRACUM Metadata Repository. Appl Clin Inform. 2021 Aug;12(4):826-35. PMID: 34433217. doi: 10.1055/s-0041-1733847.

10. Fu S, Wen A, Schaeferle GM, Wilson PM, Demuth G, Ruan X, et al. Assessment of Data Quality Variability across Two EHR Systems through a Case Study of Post-Surgical Complications. AMIA Jt Summits Transl Sci Proc. 2022;2022:196-205. PMID: 35854735.
